# Former Pupils and Supscriptions to the Journal

**Published:** 1880-08

**Authors:** 


Former Pupils and Subscriptions to the Journal.—In a recent issue we invited our former pupils—physicians—to aid us in the extension of our circulation by sending us the addresses of their former colleagues and class-mates, whom we desired should become subscribers to the "Independent Practitioner," and omitted entirely to extend a similar invitation of the dentists who were educated in part by our junior editor, whilst he was a professor in the old Baltimore College of Dental Surgery. The invitation is now made with the earnest hope that they too, will join heartily in the work of increasing our already respectably large list of subscribers.

